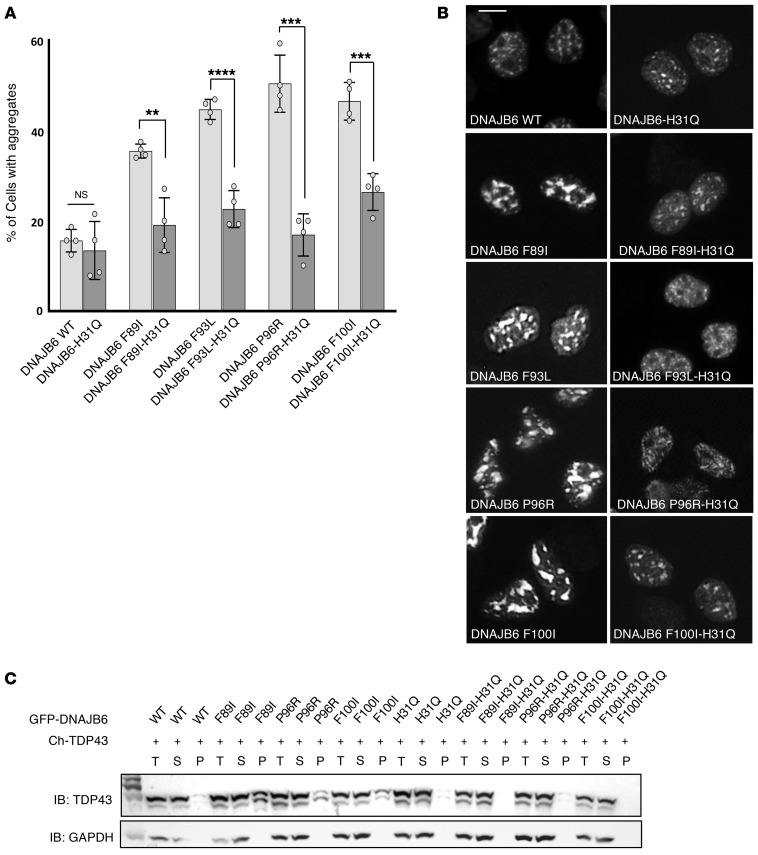# Inhibition of DNAJ-HSP70 interaction improves strength in muscular dystrophy

**DOI:** 10.1172/JCI194757

**Published:** 2025-05-15

**Authors:** Rocio Bengoechea, Andrew R. Findlay, Ankan K. Bhadra, Hao Shao, Kevin C. Stein, Sara K. Pittman, Jil A.W. Daw, Jason E. Gestwicki, Heather L. True, Conrad C. Weihl

Original citation: *J Clin Invest*. 2020;130(8):4470–4485. https://doi.org/10.1172/JCI136167

Citation for this corrigendum: *J Clin Invest*. 2025;135(10):e194757. https://doi.org/10.1172/JCI194757

In Figure 2B of the original article, an incorrect image was included for the DNAJB6 WT sample, which was an inadvertent duplication and rotation of the image for the DNAJB6 F93L-H31Q sample. The corrected figure, based on the original source data, is provided below. The HTML and PDF versions of the paper have been updated.

The authors regret the error.

## Figures and Tables

**Figure 2:**